# Modeling the Emergence of Whisker Direction Maps in Rat Barrel Cortex

**DOI:** 10.1371/journal.pone.0008778

**Published:** 2010-01-22

**Authors:** Stuart P. Wilson, Judith S. Law, Ben Mitchinson, Tony J. Prescott, James A. Bednar

**Affiliations:** 1 Institute for Adaptive and Neural Computation, University of Edinburgh, Edinburgh, United Kingdom; 2 Active Touch Laboratory, University of Sheffield, Sheffield, United Kingdom; University of Sydney, Australia

## Abstract

Based on measuring responses to rat whiskers as they are mechanically stimulated, one recent study suggests that barrel-related areas in layer 2/3 rat primary somatosensory cortex (S1) contain a pinwheel map of whisker motion directions. Because this map is reminiscent of topographic organization for visual direction in primary visual cortex (V1) of higher mammals, we asked whether the S1 pinwheels could be explained by an input-driven developmental process as is often suggested for V1. We developed a computational model to capture how whisker stimuli are conveyed to supragranular S1, and simulate lateral cortical interactions using an established self-organizing algorithm. Inputs to the model each represent the deflection of a subset of 25 whiskers as they are contacted by a moving stimulus object. The subset of deflected whiskers corresponds with the shape of the stimulus, and the deflection direction corresponds with the movement direction of the stimulus. If these two features of the inputs are correlated during the training of the model, a somatotopically aligned map of direction emerges for each whisker in S1. Predictions of the model that are immediately testable include (1) that somatotopic pinwheel maps of whisker direction exist in adult layer 2/3 barrel cortex for every large whisker on the rat's face, even peripheral whiskers; and (2) in the adult, neurons with similar directional tuning are interconnected by a network of horizontal connections, spanning distances of many whisker representations. We also propose specific experiments for testing the predictions of the model by manipulating patterns of whisker inputs experienced during early development. The results suggest that similar intracortical mechanisms guide the development of primate V1 and rat S1.

## Introduction

Mammalian sensory cortex is organized firstly by modality, and secondly into topographic maps of the corresponding sensory apparatus. The prototypical example is the map of the retina in primary visual cortex (V1). Within this retinotopic map, finer scale feature maps have been found, such as for the motion direction of visual stimuli, with nearby neurons responding to similar directions [1], [2].

Direction maps in ferret V1 emerge postnatally, and are sensitive to early visual experience [3], [4], suggesting that they result from a self-organizing process driven by visual input. Map self-organization has been modeled using networks of neurons that develop receptive fields (RFs) by Hebbian learning of correlations between input and cortical activities [5]–[7]. In such models, a balance between intracortical excitation and inhibition ensures the emergence of RFs that collectively cover the full range of motion directions; essentially, the neurons compete to respond to directions in the visual scene.

**Figure 1:**
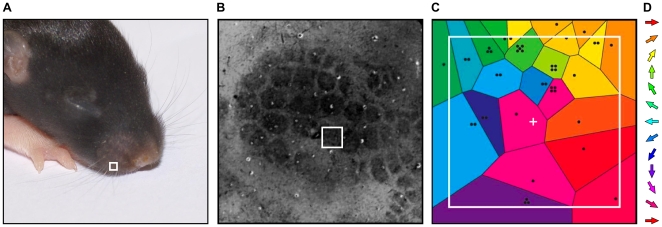
Maps in the rat whisker-barrel system. **A** The whiskers are arranged on the snout of a 10 day old rat pup in an orderly grid pattern. **B** This pattern is reproduced in barrel clusters, revealed here in a tangential section in L4 barrel cortex stained for cytochrome oxidase, such that neurons in each cluster respond preferentially to stimulation of the whisker in the corresponding position in the whiskerpad. **C** Within a supra-barrel, a pinwheel map has been measured for the direction in which the corresponding whisker is deflected [8]. The map is described as somatotopic because deflecting the principal whisker (PW) in the direction of an adjacent whisker on the snout selectively activates neurons in the PW's barrel that are closest to the adjacent whisker barrel. Reprinted and adapted by permission from Macmillan Publishers Ltd: Nature Neuroscience [8], copyright 2006; colors show the direction tuning of neurons in each location within a barrel, according to the color key in **D**. The black dots show positions of electrode penetrations, where multiple dots correspond to multiple-unit recordings. The white box in **A** outlines the base of the PW for the corresponding barrel outlined in **B** and whose supra-barrel is enlarged in **C**.

Direction maps in both real and simulated V1 are punctuated by pinwheels, where all directions are represented continuously around a central point. A similar pinwheel map has recently been measured in rat primary somatosensory cortex (S1) for the direction of deflection of the rat's whiskers [8], [9]. Andermann & Moore [8] found a pinwheel map of directions spanning the domain of layer 2/3 (L2/3) neurons most responsive to one principal whisker (PW). This domain will henceforth be referred to as the supra-barrel region or just the supra-barrel, as it is located above the L4 ‘barrel’ structure which receives thalamic input primarily from the PW. The map is somatotopically aligned to echo the overall pattern of barrels: deflection of whisker A towards whisker B evokes the strongest responses in neurons of whisker/supra-barrel A that are nearest to whisker/supra-barrel B (see Fig. 1).

The map was measured by multi-unit tetrode recordings in approximately three-month-old rats [8] but was not found in a subsequent study that used two-photon calcium imaging and rats aged approximately one month [10]. These two studies used different methods, besides the age of the animals tested and the recording techniques employed, and so the differences in their findings remain controversial (see Discussion). However, recent two-photon calcium imaging data have measured a similar map in three-month-old but not in three-week-old rats (Leger J-F., Kremer Y. & Bourdieu L., 2009, Society for Neuroscience abstract 174.13). These findings together suggest that the map for whisker deflection direction emerges during post-natal development (see Discussion). Here we explore the idea that the development of the map is driven by input from the whiskers, much as V1 feature map development is thought to be driven by input from the eyes.

Because the mapping of whisker deflection direction within the individual supra-barrel is aligned with the overall layout of the barrels themselves (see Fig. 1b), we hypothesize that it is driven by tactile experiences in which the direction of the individual whisker deflection is correlated with the stimulation of adjacent whiskers. We have previously shown that when freely moving rats explore surfaces, they make contacts on a subset of whiskers [11], [12]. Here we show in simulation that when (and only when, within the constraints of our modelling framework) the subset of deflected whiskers is consistent with the direction in which each whisker is deflected, a direction map robustly self-organizes into a somatotopic pinwheel in each supra-barrel.

## Methods

### A Model of the Barrel Cortex

We developed a model based on LISSOM (Laterally Interconnected Synergetically Self-Organizing Map [13], [14]), with afferent projections that are constrained to simulate those from the layer 4 (L4) barrels to the supra-barrels in L2/3. The model was built using the Topographica simulator [15], which is freely available at www.topographica.org.

**Figure 2:**
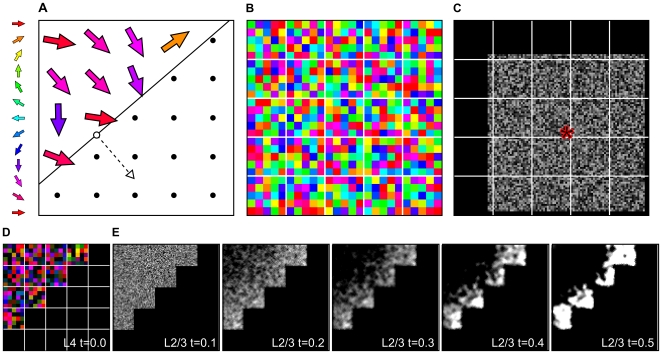
Model diagram and activity before any learning. **A** 25 whiskers are arranged in a regular grid, where some are deflected (colored arrows) and some are not (dots). Deflected whiskers are those impinged by a wide stimulus (solid line) moving in the direction of the dashed line and unfilled arrow (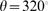). The stimulus is a half plane, which has moved almost half-way through the whisker field in this example. Deflected whiskers are those to the left of the plane. Impinged whiskers are deflected roughly in the direction of stimulus motion, but we apply normally distributed noise to each, with concentration parameter 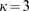 in the example. **B** The L4 sheet is divided into barrels (delineated by white), each containing 25 neurons with pre-assigned MEDs (pixel color) from around the circle, and located arbitrarily within the barrel. **C** L2/3 is divided into supra-barrels (21
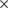
21 neurons in each), such that each neuron receives weighted projections from all L4 neurons in the corresponding barrel. Each L2/3 neuron also receives fixed excitatory lateral connections from itself and its 8 immediate neighbors (its lateral excitatory connection field). Each also receives inhibitory connections from all neurons that fall within a 4
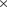
4-barrel area (84
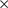
84 neurons) centered on its location; the lateral inhibitory connection field for the neuron marked * is shown. The brightness indicates connection strengths from * to each neuron before training. **D** The example input is represented in L4 by activating neurons whose MEDs are similar to the direction of deflected whiskers. **E** Initially random activity in stimulated L2/3 supra-barrels migrates to the leading edge of the stimulus as lateral interactions settle for each of steps 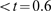. All plots are normalized separately.

The model comprises twenty-five whiskers arranged into a 5
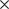
5 grid, or ‘whisker field’ (Fig. 2a), 25 corresponding ‘barrels’ in L4 S1 (Fig. 2b), and a sheet of 105
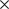
105 L2/3 neurons (Fig. 2c). Each barrel contains 25 directionally tuned afferent units that code for the stimulation of each whisker. Based on the afferent connections from L4, L2/3 can also be divided into a 5
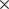
5 grid of ‘supra-barrels’. There are 21
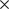
21 neurons in each supra-barrel, such that neurons located in each receive input from the L4 units coding for stimulation of the corresponding isomorphic (principal) whisker.

We first give a general overview of how the model works. An input pattern represents how the 5
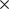
5 grid of whiskers interacts with a tactile stimulus, determining whether each whisker is deflected and in what direction. This pattern is then encoded as a pattern of activation in L4. When the pattern is presented to the network, activity propagates from the L4 barrels (see Fig. 2d) to the corresponding L2/3 supra-barrels (see Fig. 2e), via weighted connections whose strengths are initially set to random values. L2/3 neurons then interact laterally, through recurrent connections that are net excitatory over very short distances and inhibitory over very large distances. Lateral interactions are allowed to stabilize through a number of settling steps, focusing the initial L2/3 response into discrete bubbles of activity across L2/3 (as in Fig. 2e). Once the lateral interactions have settled, afferent and lateral inhibitory weights are updated with a Hebbian learning rule, activation is reset to zero, and a new stimulus is presented to the network. The next four Methods sections describe these steps in detail.

### Stimulating the Whiskers

Each whisker 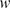 is assigned a coordinate spaced on a rectangular grid such that horizontally and vertically adjacent whiskers are 1.0 units apart, and diagonally adjacent whiskers are 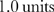 apart. The layout of the whiskers on the grid is illustrated in Fig. 2a. To construct each input pattern, we choose a linear boundary passing through a random point 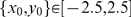 and with outwardly-pointing normal in a random direction 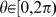. Whiskers inside the boundary are deflected, and those outside are not.

In line with our hypothesis that a correlation between whisker direction and the overall pattern of activated whiskers could align maps of whisker direction, we define the perfectly correlated direction for each whisker deflection to be 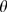. We can then control the strength of this correlation by drawing individual deflections randomly from a distribution centered on 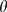. We use a circular normal distribution (a Von Mises distribution; see [16]) and vary its concentration parameter 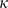. This is shaped like a normal distribution for 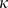 values between 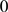 and 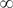, but at 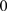 the distribution is flat, and 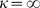 describes a delta function. For example, when 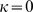 the whiskers would each be deflected in random directions, and when 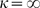 they would each be deflected at 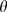. See Fig. 2a for an illustration of this process.

This model is a simple abstraction of the complex (and largely unknown) pattern of whisker–stimulus interactions present during early development, focusing only on the assumption that local subsets of the whiskers are usually impinged by large stimuli moving from outside to inside the whisker field. Such stimuli might be, for example, the floor and other surfaces in the environment, a littermate's foot, tail or head, or a part of the mother's body. For clarity in the remaining sections, when we refer to a direction of motion, we mean the motion of a stimulus relative to stationary whiskers, not that of the whiskers due to locomotion or active whisking behavior. Even so, note that both types of motion would yield the same relative motion, and thus indistinguishable patterns of activation in the model.

### Activating the Barrels

Neurons located within a rat L4 barrel are tuned to the direction in which the PW is deflected [17], [18]. Although neurons with similar maximally effective directions (MEDs) are clustered together, evidence for a systematic spatial arrangement of these domains in L4 is weak [8], [9]. L4 MEDs are consistent throughout post-natal development [19], and neither the location nor directionality of the neuron is known to predict adjacent-whisker effects [20], [21].

Accordingly, each afferent unit (i.e., each L4 unit) 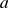 is pre-assigned a fixed MED for deflections of the PW, chosen randomly from 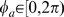. We use a cosine curve scaled to reflect the broad directional tuning of L4 neurons:
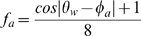
(1)where the firing rate 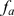 of each L4 unit increases when the PW is deflected in a direction more similar to its preferred direction.

### Lateral Interactions

Following deflection of a single rat whisker, excitation is relayed through corresponding groups of neurons in rat brainstem and thalamus to the isomorphic L4 barrel. Excitation then projects into the supra-barrel in L2/3, and subsequently spreads across L2/3 into adjacent domains [22]. However, the overall long-distance effect of a strong whisker deflection is inhibitory, perhaps due to disynaptic inhibition. For example, Derdikman et al. [23] measured a consistent difference-of-Gaussians profile of activity across L2/3, in which inhibitory effects range significantly further across adjacent supra-barrels than excitatory effects, for the duration of the response following PW deflection.

Studies in which adjacent whiskers are sequentially deflected also reveal strong suppression of responses to the second whisker by prior deflection of the first [20], [24]–[26], and the same has recently been demonstrated for stimuli that involve many whiskers [27], [28]. Interestingly, cross-whisker suppression is maximal at the time-scale measured as the mean interval experienced by rats trained to whisk into a stimulus (approximately 20ms) [29].

With these observations in mind, we set up model L2/3 neurons to receive excitatory connections from themselves and the eight immediately adjacent neurons, so that the activity of the pre-synaptic neuron increases the response of adjacent post-synaptic neurons. Over this range and over greater lateral distances (a square area four supra-barrel widths across), neurons receive inhibitory lateral connections.

Note that these connections implement the observed net pattern of lateral interactions, and as described in the Discussion, do *not* represent any assumptions about the relative lengths of actual inhibitory and excitatory lateral connections in S1.

It is plausible that L2/3 neurons receive feed-forward input arising from multiple whiskers. However for simplicity in the model the twenty-five units of each L4 barrel all project to each of the 441 neurons in the isomorphic supra-barrel only. Hence we model the connectivity from barrel to supra-barrel as all–to–one. The lateral excitatory and inhibitory connection fields are not restricted by the barrel borders imposed on the afferent projection from L4, but are instead centered on the location of each cortical neuron (as suggested by evidence from [10], [30]–[33]; see example in Fig. 2c). Before training, the weights in the connection fields for each L2/3 neuron (afferent, excitatory and inhibitory) are uniform random values, normalized to sum to 1.0 in each connection field.

Following the reduced LISSOM model [14], the activity 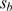 for a L2/3 neuron at location 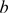 is the weighted sum of the activity in the corresponding barrel:
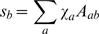
(2)where 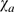 is the activation of afferent neuron 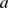 in the barrel projecting to cortical neuron 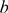 and 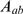 is the corresponding afferent weight. After the initial response of a cortical neuron is calculated, activation propagates laterally across L2/3 for 9 settling steps; little change in the activation patterns is observable after 5 steps. Lateral interactions affect the activity 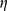 of a single cortical neuron 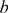 according to:
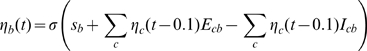
(3)where 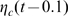 is the activity of another L2/3 neuron 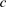 during the previous settling step, 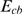 is the excitatory lateral connection weight from that neuron to neuron 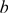, and 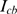 is the inhibitory connection weight. The activity is squashed through 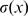, a piecewise-linear approximation to a sigmoidal activation function:
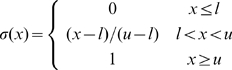
(4)where 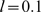 is a lower-bound threshold and 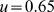 is the upper bound, i.e., the saturation point of the (linearly approximated) sigmoidal region. The values for all of these parameters were determined in pilot work so that the network would group activity into bubbles on the approximate spatial scale of the supra-barrel (see example in Fig. 2e).

### Learning

After settling, both afferent and lateral weights are updated via a Hebbian learning rule with divisive normalization:
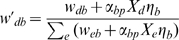
(5)where 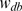 is the current afferent or lateral connection weight from 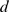 to 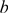, 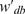 is the value of the weight to use in the next input presentation, 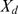 is the pre-synaptic activity after settling, and 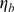 is the activity of neuron 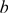 after settling. For unit 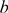, 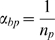 is the Hebbian learning rate for connections of type 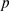 (either afferent, 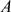, or lateral inhibitory, 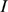), where 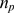 is the number of neurons in the connection field for neuron 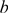. For example, 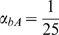, as there are 25 afferent units in the afferent connection field (or barrel) connecting to each L2/3 neuron 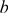. The afferent and lateral inhibitory connections are normalized separately. We note that by using a divisive rather than subtractive normalization, weights are redistributed rather than driven to saturation after each training pattern; for a detailed discussion of this behavior see [34]. This process of input presentation, activation, settling, and learning is repeated for each of 5,000 random input patterns.

## Results

### Activity Bubbles Migrate to the Leading Edge of the Stimulus

When the very first stimulus is presented to the model (Fig. 2d), activity first propagates from the barrels associated with deflected whiskers to layer 2/3, exciting each neuron in the isomorphic supra-barrels randomly (Fig. 2e, t = 0.1). L2/3 neurons then begin to interact laterally (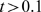), each becoming more active if it is similar to its immediate neighbors and dissimilar to more distant neighbors, and less active otherwise. This process continues as the network settles, and as larger groups of activity merge they migrate toward regions of least net inhibition. Hence, bubbles of activity form at the high-contrast edges of the supra-barrels that correspond to whiskers located furthest forward in the direction of the stimulus. By furthest forward we mean those inside the linear boundary that are closest to it, and hence those whiskers that would have been deflected most recently by contact with the stimulus. If the direction in which the whiskers are deflected is consistent with the orientation of the stimulus, then neurons in these regions of the supra-barrels will learn to become associated with the L4 neurons that encode the somatotopically consistent direction of whisker deflection.

As an example, a stimulus boundary moving upwards would be oriented so as to bisect the whisker field through one of the whisker rows. It would deflect all whiskers located within and below that row in an upwards direction, and would preferentially activate L4 units representing upwards deflections. Activity in L2/3 would migrate to the top portion of the supra-barrels in the same row, and these neurons would learn stronger weighted connections to the active L4 units representing upwards deflections.

Repeated for stimuli whose leading edges bisect all points in the whisker field, at all orientations, this process will bias the network to arrange direction preferences somatotopically in each supra-barrel.

### A Somatotopic Pinwheel Emerges in Each Supra-Barrel

**Figure 3:**
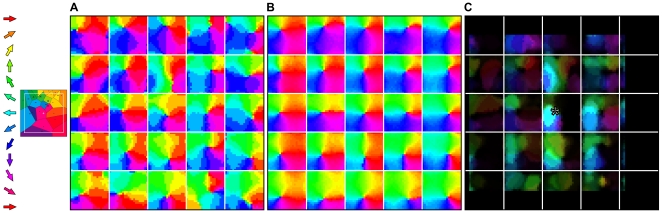
A somatotopically aligned map of whisker deflection direction emerges in each supra-barrel. **A** Example map from one network trained on 5,000 input patterns in which whisker deflection directions are each concentrated towards the orientation of the stimulus (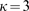). Maps in each supra-barrel are a match to that measured by ref. [8] in which neurons on the left of each supra-barrel, for example, prefer leftward deflections of the PW. Reprinted and adapted by permission from Macmillan Publishers Ltd: Nature Neuroscience [8], copyright 2006. Supra-barrels are delineated by white lines. **B** Mean direction preference for neurons at each cortical location, over the 20 networks in the same data set, showing that the organization is consistent across runs. **C** Plot of the long range lateral connection strengths, from the representative example neuron at the position marked by *, to the rest of the cortical map. Pixel brightness indicates lateral weight strength, and the color indicates the preferred deflection direction of each connected neuron. This neuron becomes most strongly connected to others, some located many supra-barrels away, that are tuned to similar directions of PW deflection.

For each value of 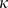 = 0, 1, 2, 3, 4, 5, and 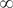, 20 networks with different random initial weights were trained on different sets of 5,000 random input patterns; a total of 140 simulations were run. As a reminder, larger values of 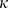 increase the concentration of the individual whisker deflection directions towards the movement direction of the stimulus (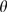). Once the process of self-organization was complete, direction map plots were measured by deflecting each whisker through 16 directions, and then coloring each L2/3 neuron by the deflection direction that evoked the largest response. Lateral interactions and learning were turned off during this process. We note that once some learning has taken place, direction maps based on the feed-forward response are almost indistinguishable from those based on the activity after settling. We report maps based on the feed-forward response as it can be calculated more quickly for the large numbers of simulations used, and so as not to reveal an arbitrary mapping in networks that have received no previous input. An example map measured from one network trained on 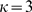 inputs is shown in Fig. 3a.

For the 20 networks run at each value of 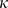, we constructed plots of the mean preferred deflection direction at each cortical location. For 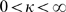, these plots revealed a somatotopically consistent pinwheel spanning each supra-barrel; Fig. 3b shows such a plot for the 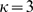 maps. Each is a qualitative match to that measured by [8] in L2/3 barrel cortex. Notice that the center of the pinwheel is shifted in each supra-barrel away from the center of the cortex. This reflects an implicit bias for deflections of the PW to occur more often towards the center of the whisker field, because the origin of the stimulus was confined to fall in a space not much larger than that occupied by the whiskers.

Similar plots for the control 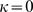 reveal no global alignment, suggesting that a somatotopic relationship between the deflection direction and the combination of deflected whiskers is required to organize directional preferences somatotopically. Suprisingly, when the correlation is perfect (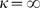), map organization does not become consistent with the somatotopic ideal. Inspection of the individual maps suggests that 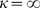 networks instead tend to maximize continuity of directional preferences across the entire sheet, without respecting the boundaries between supra-barrels (see below).

### Connections Between Similar Directions and Different Whiskers

Because the Hebbian rule strengthens connections between correlated neurons, we might expect the final patterns of long-range lateral connections to reflect the fact that even distal whiskers are deflected in similar directions. Such an effect is clear in an example L2/3 map in which pixel brightness is scaled by the strength of the weights to one neuron from the rest of the sheet (Fig. 3c). The example neuron prefers leftward (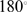) deflections of the central whisker and becomes connected most strongly to neurons in L2/3 that also prefer leftward deflections of their PWs. Overall, we found a significant correlation between the strength of the lateral inhibitory weight between each pair of L2/3 neurons and the absolute difference between their preferred deflection directions (mean Pearson's 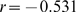, range 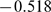 to 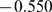, across 20 networks each trained on 5,000 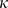 = 3 inputs). Hence, the model predicts connectivity in L2/3 between patches of directionally consistent neurons with different PWs. Notice also that connection strength is greatest between neighboring neurons within the barrel, and falls off with the distance to the pre-synaptic cell (see [10]).

These findings are consistent with those from experiments showing the strongest lateral interactions when whiskers are sequentially deflected in similar directions [24], [26]. Similarly, in tree shrew V1, long-ranging connections have been found to connect neurons that respond to similar orientations of visual stimulus [35].

### Input Correlation Improves Pinwheel Alignment but Not Quality

To quantify our observations, we analyzed the direction maps per supra-barrel with reference to an ideal somatotopic pinwheel template, defined for each neuron as the angle of its location from the center of each supra-barrel. More formally, each L2/3 neuron was assigned a coordinate (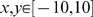) with respect to the supra-barrel center, and its preferred deflection direction according to the template was defined using the quadrant-specific arctangent function *atan2* 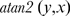. The template value at the origin is undefined so the neuron at each supra-barrel center was discounted from further analyses.

**Figure 4:**
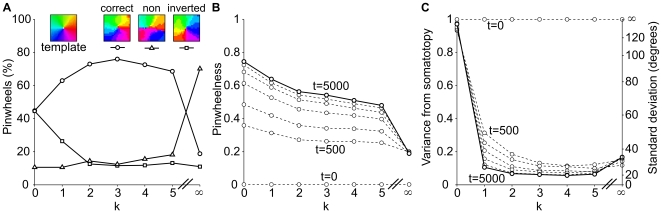
Analysis of pinwheel quality and somatotopic alignment per supra-barrel in 20 model networks. **A** At t = 5,000, direction maps in each supra-barrel were compared to the template pinwheel (inset) and classed as somatotopically correct pinwheels (the example map has a ‘pinwheelness’ score of 0.9), somatotopically inverted pinwheels (example score -0.9) or not pinwheels (score 0.2), as described in Results. When there is no correlation between the direction in which each whisker is stimulated during training (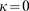), pinwheel maps emerge in each supra-barrel, but they are equally likely to rotate clockwise or counter-clockwise. When such a correlation is present in the inputs (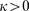), the number of supra-barrels containing pinwheels that rotate in a somatotopically consistent way increases to a maximum of 76%. Surprisingly, perfectly correlated inputs (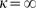) degrade pinwheel quality. **B** This behavior is reflected in a plot of absolute ‘pinwheelness’ scores, in which all but the scores for 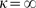 progress over training iterations (t = 0, 500, 1,000, 2,000, 3,000, 4,000 in progressive dashed lines) toward good scores at t = 5,000 (solid line). Scores are highest for 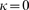, suggesting that networks trade a bias to maximize pinwheelness for one towards somatotopic alignment as 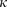 is increased. **C** shows that pinwheels rotating in the correct direction become aligned to the somatotopic template, with a final circular standard deviation 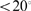 for 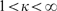.

An angular-angular correlation between the measured map and the template gives a score of the correspondence between the two that is rotation independent, and the absolute value of this quantity is also independent of clockwise and counter-clockwise orientation around the supra-barrel center. We can therefore define *pinwheelness* as the magnitude of the angular-angular correlation coefficient. For the 500 supra-barrel maps (20 networks times 25 supra-barrels) at each value of 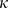 = 0, 1, 2, 3, 4, 5, and 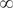, we first counted those with counter-clockwise or clockwise orientation with a correlation coefficient greater than that measured in barrel cortex (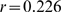; ref. [8]). We classified supra-barrel maps wherein 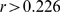 as rotating counter-clockwise about the supra-barrel center and therefore somatotopically correct, those where 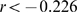 as clockwise and thus somatotopically inverted, and where 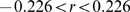 as non-pinwheels (see Fig. 4a). At 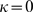, 90% of 500 supra-barrels developed pinwheels, but these were equally likely to be oriented clockwise or counter-clockwise. For 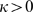, the number of pinwheels that rotate counter-clockwise around the supra-barrel increases to a peak of 76% at 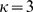. However, when inputs had a perfect alignment between whisker deflection direction and the orientation of the edge of the stimulus (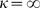), the number of well-defined pinwheels dropped to just 30%.

These trends are reflected in a plot of absolute pinwheelness (Fig. 4b), which is notable because it shows maximal pinwheelness when 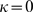. Hence, even without a consistent somatotopic relationship between the whiskers, the supra-barrels still discover the circular topology of the space of possible deflection directions, communicated by the coactivation of L4 cells with similar MEDs.

The overall trend is for pinwheelness to decrease as 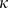 is increased. Thus an increase in somatotopic information in the inputs does not create pinwheels, but only aligns them somatotopically. This is confirmed in a plot of the circular standard deviation between the counter-clockwise supra-barrel maps and the template (Fig. 4c), which shows a distribution all the way around the circle for 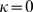 (std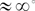) which decreases to 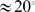 when whisker deflection direction and location are well correlated during training (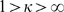).

### Biased Whisker Inputs Create Anisotropic Maps

**Figure 5:**
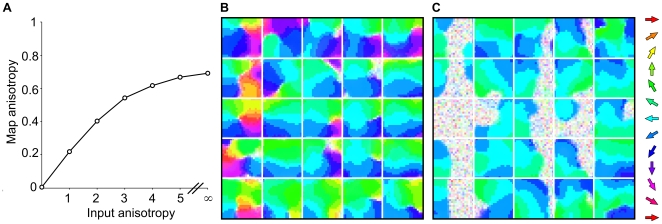
Anisotropic inputs create anisotropic maps. Values of 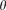 were drawn from circular normal distributions with varying degrees of concentration (input anisotropy), towards a mean of 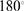. Results suggest that biased experience to a particular direction of stimulus will cause an over representation of that direction in the supra-barrels. Map anisotropy scores converge to 0.69 (out of a maximum of 1.0) when the networks are trained in a regime where half-plane stimuli always move in the same direction. **B** shows an example map from a network trained on input anisotropy 3.0, where pixel saturation indicates a lower direction selectivity for each neuron. Distorted pinwheel structures still form in many barrels, but the map is clearly dominated by neurons preferring 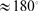 deflection directions. **C** shows a similar map from a network trained on input anisotropy 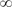, wherein patches of non-selective neurons form on the right side of the left most supra-barrels where the leading edge of the stimulus is least likely to occur.

Next we tested how a statistical bias in the distribution of 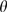 might affect map organization (see Fig. 5). This is important to consider because biases in the representation of certain deflection directions have been found in the barrel cortex of the adult rat (see Discussion). To this end we ran networks for 5,000 input patterns, this time drawing 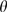 from a circular normal distribution with mean 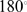. Here the concentration parameter of the distribution serves to control input pattern anisotropy, where zero anisotropy means that 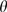 is drawn uniformly from around the circle. In addition, we ran 20 different networks each per input anisotropy value 1, 2, 3, 4, 5 and 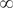 (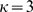). Hence, for networks in subsequent conditions, the movement of the half-plane stimulus was more likely to be around 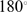.

To quantify the effects of the bias (Fig. 5a), we summed the vectors corresponding to the preferred direction of each neuron trained under a given bias. The averaged length of this resultant vector gives a score of how concentrated the direction preferences are towards one direction, and hence provides a score of map anisotropy. A map anisotropy score of zero indicates that maps represent directions isotropically, whereas a maximum score of 1.0 indicates that the map is comprised of neurons that all prefer the same direction.

We found that as the bias for 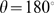 input patterns increased, so did the proportion of neurons whose preferred direction became aligned towards 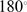 (mean preferred directions ranged 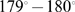 for maps trained with a bias). The trend converges to a map anisotropy score of 0.69 out of 1.0 when 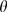 is always 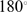, which is less than 1.0 owing to the broad and fixed direction tuning of the L4 input units and the 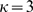 noise applied to the individual whisker deflection directions.

For input anisotropies up to 4, the biased maps themselves still organize to represent a range of directions around 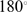 continuously, in a distorted pinwheel local to each supra-barrel (Fig. 5b). Above 4, some patches opposite the biased orientation remain un-selective throughout training, because very few 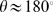 input patterns will create a leading edge effect to drive bubbles of activity to the opposite edge of the supra-barrels (Fig. 5c).

Thus the model predicts that strong biases in the distribution of experienced deflection directions will be reflected in the direction maps, both as expanded regions for over represented directions, and as patches of less selective neurons in the somatotopically correct locations for under-represented directions.

### Maps Do Not Organize Somatotopically without a Correlation between Whisker Combination and Deflection Direction

**Figure 6:**
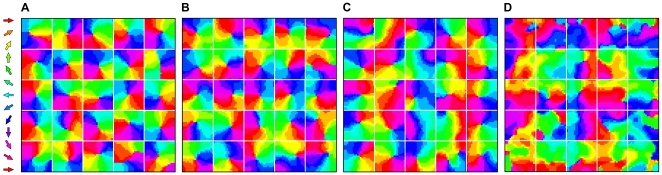
Model maps organized in control experiments and at 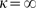. **A** Whisker deflection directions are independent of one another. Example direction map from a representative 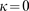 network, which develops good pinwheels in each supra-barrel but no consistent global organization. **B** Removing global correlations. Example map measured from a network trained on 5,000 inputs wherein the location of the stimulated whiskers was randomly shuffled on each iteration (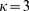). **C** Direction map measured from one representative network trained on 5,000 inputs wherein the whiskers are deflected in the same combinations as in the normal case, but the mean direction in which they are deflected bears no relation to the stimulus direction implied by this combination (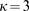). In both controls, maps resemble V1 orientation or direction maps rather than rodent S1 maps, because they cover all directions continuously on the local scale but have no consistent global alignment. **D** When whisker deflection directions are perfectly correlated with the whisker combination (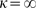), the supra-barrel borders no longer affect the input correlations, and so the map groups similar directions together rather than developing independent pinwheels.

We have already examined the results of the first control condition, the case where 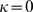, in which we see good pinwheel maps form in each barrel but no consistent global alignment (example in Fig. 6a). The networks were then trained in two additional control conditions (both at 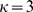).

In the second control (Fig. 6b), the location (but not the number) of the activated whiskers was randomly permuted for each input pattern. For example, the stimulus shown in Fig. 2d would be reconstructed so that a random subset of ten whiskers were deflected. The activated whiskers were distributed randomly over the twenty-five possible locations on the whiskerpad and were therefore not confined to any particular region of it. Hence the global information about somatotopy was removed from each input pattern, but the level of afferent activation and the consistency between the directions in which the whiskers were deflected remained. Maps organized in this condition developed reasonably strong pinwheels, but again had no global alignment (standard deviation from the template 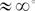). Instead, they organize more locally to be similar to primate V1 maps for orientation or direction, becoming composed of continuous regions that are punctuated by pinwheel, linear and saddle-point discontinuities (see ref. [14]), largely ignoring the barrel boundaries.

In the third control (Fig. 6c), the stimulus deflected whiskers in the same combinations as in the main simulation, and for each stimulus whiskers were deflected in similar directions (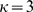). However, the mean of the distribution from which each deflection direction was drawn was random and independent of the orientation of the stimulus. Hence whisker deflection directions were again correlated with one another but unrelated to the global direction implied by the combination of activated whiskers. Again, direction maps that emerge in this control condition are more similar to primate V1 maps than rodent S1 maps because they have no overall somatotopic organization.

These results confirm that only when the overall pattern of deflected whiskers correlates with the direction in which each whisker is deflected, do somatotopic direction maps self-organize consistently within each supra-barrel.

### Experimental Manipulations

**Figure 7:**
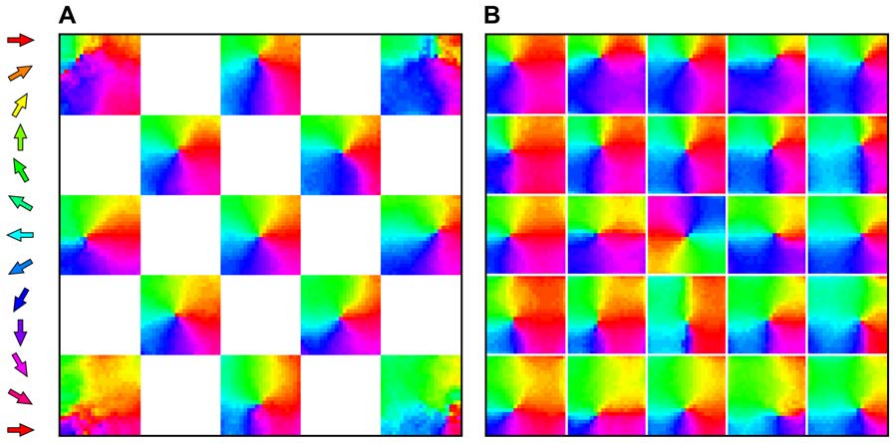
Predicting mappings for experimentally manipulated whisker inputs. **A** Whisker trimming experiment. Whiskers in a chessboard configuration of the model barrels were deprived of whisker input. The plot shows the mean directional preference over 20 networks. Neurons in deprived supra-barrels have no opportunity to learn connections to particular L4 neurons. However, spared supra-barrels are still able to form reasonable somatotopic pinwheel maps. Thus the model does not predict any specific reorganization of spared portions of the map for the isolated whisker trimming case. **B** Anti-correlated whisker experiment. If a central whisker is consistently deflected in the direction opposite its neighbors, neurons in the central barrel should develop RFs for deflection directions opposite those suggested by their somatotopic location, forming a somatotopically inverted pinwheel in the corresponding supra-barrel. The mean preferred direction for neurons at each location is plotted (N = 20 different networks). This prediction could be tested by training rats on artificial stimuli in which the central whisker is deflected, for example, rostrally (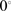) whenever the more caudal whiskers are primarily deflected, during the critical period. Although difficult to perform, this experimental paradigm would be very useful for assessing the time course of map plasticity.

Computational models, like other theoretical formulations, should make specific predictions that can be tested through experimentation. Two such predictions, arising from the current work, are illustrated in Fig. 7.

For the first prediction, we simulated a whisker-trimming experiment by depriving whisker input to a chessboard configuration of the barrels [36] (see Fig. 7a). Although no prediction can be formulated about the organization of maps in deprived supra-barrels, somatotopically aligned maps emerge in the spared supra-barrels. Thus the model predicts that isolated whisker trimming even early in development will not have a significant effect on the development of pinwheels in the supra-barrels for the remaining whiskers. Only when enough whiskers have been trimmed to isolate a supra-barrel from those that interact laterally with it, will somatotopic alignment be disrupted.

The second prediction is that if a central whisker is consistently deflected opposite the direction of its neighbors, the organization of direction preferences in the corresponding supra-barrel will be a pinwheel that is somatotopically inverted (see Fig. 7b). In other words, deflections of whisker A towards whisker B will evoke the strongest responses in supra-barrel A neurons located furthest from supra-barrel B. With the advent of apparatus capable of independently stimulating up to twenty-five whiskers [27], [28], [37], the anti-correlated pinwheel experiment could now be undertaken with very precise control.

## Discussion

We have demonstrated how a computational model of L2/3 barrel cortex can develop a map of whisker deflection direction that is a strong qualitative match to that measured in the rat barrel cortex by Andermann & Moore [8]. The main finding is that pinwheel maps of whisker deflection direction align somatotopically in each simulated supra-barrel. Thus the somatotopic pinwheel map should emerge across all supra-barrels provided that (i) net L2/3 interactions concentrate activity into bubbles smaller than a supra-barrel, (ii) these bubbles migrate to areas corresponding to the leading edge of a tactile stimulus, (iii) whiskers are consistently deflected away from stimuli.

The two key assumptions of the model, which need to be validated with further experimental work, are as follows. First, the model assumes that whisker contacts experienced by young rats correlate whisker combination with whisker deflection direction. Second, it assumes that the lateral extent of net excitatory interactions is less than that of net inhibitory interactions in barrel cortex, regardless of the detailed circuitry that implements these interactions. The two key predictions of the model, for normally developed barrel cortex, are as follows. First, supra-barrels for all of the large whiskers will contain a somatotopically aligned pinwheel map of PW direction, although pinwheel centers may be shifted for more peripheral whiskers. Note that only the direction map for a central supra-barrel has been established to date [8]. Second, L2/3 neurons with similar directional tunings will be synaptically coupled, certainly with neighbors in the supra-barrel, and perhaps with those located several supra-barrels away. These predictions and the two key assumptions are testable immediately, and should not require experimental manipulation of the patterns of input to the whiskers.

In the present study, the efficacy of all whisker deflections was chosen to be equal: a whisker is either deflected or it is not. However, we could have chosen to associate different strengths to each whisker deflection, e.g. by defining a gradient of deflection strengths that decreases along the path of the stimulus. Networks trained in this way develop the same map organization as those reported (data not shown), because they essentially repeat the leading edge effect at multiple locations for each training pattern.

We chose LISSOM to model feature map development in the barrel cortex because it emphasizes lateral cortical interactions, because it produces realistic primate V1 feature maps [14], and because many comparisons have been drawn between whisker S1 and primate V1 at the level of the cortical map [38], [39]. We expect that other models (e.g. self-organizing maps or correlation-based–learning approaches) would yield similar overall map organization, if they implement similar lateral interactions. However, with the exception of the LISSOM-like model of ref. [40], alternative models do not simulate explicit, modifiable lateral weights, and so could not reveal an emergent connectivity between directional representations that span many supra-barrels (as in Fig. 3c).

It is important to emphasize that LISSOM does not require any assumption that long-range inhibitory interactions are implemented via long-range inhibitory connections in the cortex. The long-range inhibitory interactions measured in the barrel cortex by ref. [23] are presumably implemented by long-range excitation of local inhibitory neurons [32], as is thought to be the case in V1 for high contrast visual inputs (see refs. [41], [41]–[45], and see also [39]). There is now growing evidence for pervasive disynaptic inhibition in barrel cortex, at least in L4 to L2/3 circuit pathways [46]–[49]. Whether long-range inhibition is monosynaptic or disynaptic is not important for the modeling results, only that it be net inhibitory at long distances for strong deflections.

Given the robust emergence of pinwheel maps in the model, it is intriguing that although a recent two-photon calcium imaging study from Kerr et al. [10] measured similar levels of directional tuning to ref. [8], they found no evidence for a systematic map of deflection direction in L2/3. A number of methodological differences might account for these findings, such as anesthetics with different effects on intracortical inhibition [50], or weaker stimulation velocity, as suggested by ref. [51]. The differences might be reconciled by recent two-photon calcium imaging data (Leger J-F., Kremer Y. & Bourdieu L., 2009, Society for Neuroscience abstract 174.13) which report a somatotopic pinwheel organisation in three-month old rats (the approximate age of the rats of Andermann & Moore [8]) but no correlation between the location of the neuron and its directional tuning in three-week old rats (the data of Kerr et al. [10] were obtained between postnatal days 25 and 35).

It also remains to be seen why an organisation for directional tuning accounts for just a portion of the variability of supragranular neuronal responses to deflection of the whiskers (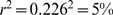, [8]). Input to the model neurons communicates only information about whisker direction, and so produces a very smooth mapping for direction in all of our simulations. However, we should assume that cortical neurons compete to represent many features of single– and multi– whisker stimuli, and so expect maps for direction to be degraded by the extent to which these additional features are described by thalamocortical input. To illustrate, consider the primary visual cortex of higher mammals, wherein each neuron participates in topographic mappings for eye preference and disparity, as well as for stimulus location, orientation, motion direction, spatial frequency, and colour. Deflection direction may not even be the best-represented feature after whisker identity, as suggested by a decrease in the information about direction carried by spikes recorded from neurons higher along the neuraxis [52]. The question of what additional, presumably higher-order, features are coded for by the activity of barrel cortex neurons remains an exciting and very open one.

There are numerous other phenomena in the whisker/barrel system that might yet be explained by Hebbian learning of whisker experience. In the paralemniscal brainstem nuclei, it has been suggested that the overrepresentation of dorsal deflections [53] may be due to the greater preponderance of dorsal deflections during rat locomotion and exploratory behavior (e.g., [12]) biasing cell receptive field properties via Hebbian learning. In the thalamus, competitive interactions between nuclei [54]–[57] might shape the direction map measured across the vertical extent of thalamic ‘barreloids’ [58], [59], and feedback to thalamic direction maps from those in infragranular cortical layers might also play a role [60]. For infragranular neurons, a correlation has been reported between selectivity for motion directions administered in waves across many whiskers, and for responses to particular adjacent whiskers [28]. This data suggests the presence of a map for wave direction that is distinct from the single-whisker direction map, and might develop in a model extended to include a representation of layer 5 (see also [27], [61], [62]). Such maps could be used by the animal to discriminate stimulus features such as orientation [63].

In the adult cortex, a number of studies have reported that activity propagates preferentially along the barrel rows compared with the arcs [23], [64], [65], that a row bias exists also in axon distributions across layer 2/3 [33], and that rostral and caudal deflection directions are overrepresented [8], [9]. These biases may reflect tendencies of adult rats to encounter objects head-on and to actively palpate the whiskers forwards and backwards, but it is difficult to determine the precise patterns of whisker deflections in live animals to use as inputs to the model. We are now beginning experiments with a mobile whiskered robot to determine what patterns of whisker deflection are common in such encounters [66], but can predict from the results of Fig. 5a that these would lead the model to expand representations of more common deflection directions in the map. We have also begun a series of experiments using robot-controlled collisions with an array of artificial whiskers to investigate the extent to which stimuli of different shapes correlate the relative position of the whisker with its deflection direction (Wilson S.P., Mitchinson B., Pearson M., Bednar J.A., Prescott T.J, 2009, Society for Neuroscience abstract 174.4).

Each of the phenomena discussed above likely involves interactions at the neural population level between multiple whisker pathways. Hence each are suitable for investigation with network models like ours, the first to explore interactions between whiskers in detail. To progress towards a complete systems-level model of multiwhisker processing, the ideas developed here can be integrated with existing models of detailed temporal processing of single-whisker events. Relevant models are available for the rat whisker [67], the follicle and ganglion [68], [69], the thalamus [70] and the barrel cortex [50], [71]–[74].

Of the existing computational models, the only one to focus on S1 direction tuning is from Puccini et al. [71]. They presented whisker-direction inputs to an integrate-and-fire neuron as differences in the latency and strength of their excitatory and inhibitory components: excitation arrives faster, and both are stronger, for whisker deflections more similar to the MED [75]. If this feed-forward model were to learn and evaluate inputs from adjacent-whisker cells, the relative contributions of feed-forward versus recurrent inhibition to constructing directional RFs could be detailed (see refs. [46], [76]). In a network of such neurons we might hope to predict how the spatial organization of direction within a supra-barrel interacts with that for alternative features, e.g. stimulus frequency [77].

The validity of our model could be tested using the anti-correlated whisker manipulation suggested in Fig. 7b. If robust changes are found to the directional RFs of L2/3 neurons, without producing an anti-correlated pinwheel, then our description of either the sensory input, or of the resulting cortical interactions, is inaccurate. On the other hand, finding an anti-correlated direction map under these conditions would be very strong evidence for input-driven self-organization as a mechanism for establishing RFs in the barrel cortex. Previous studies detailing the plasticity of cortical feature maps have shown how cortical organization can be disrupted or exaggerated by altered sensory stimuli (for example see ref. [78]), but if our anti-correlated pinwheel prediction is confirmed we could use it to ask, on what timescale could a very specific map organization be entrained: seconds, hours or days? Answering this question could help clarify the ongoing relationship between the sensory environment and the organization of cortical sensory areas.
